# Purinergic Signaling and Inflammasome Activation in Psoriasis Pathogenesis

**DOI:** 10.3390/ijms22179449

**Published:** 2021-08-31

**Authors:** Davide Ferrari, Fabio Casciano, Paola Secchiero, Eva Reali

**Affiliations:** 1 Department of Life Science and Biotechnology, Section of Microbiology and Applied Pathology, University of Ferrara, 44121 Ferrara, Italy; davide.ferrari@unife.it; 2 Department of Translational Medicine and LTTA Centre, University of Ferrara, 44121 Ferrara, Italy; fabio.casciano@unife.it (F.C.); paola.secchiero@unife.it (P.S.); 3 Interdepartmental Research Center for the Study of Multiple Sclerosis and Inflammatory and Degenerative Diseases of the Nervous System, University of Ferrara, 44121 Ferrara, Italy; 4 Department of Biotechnology and Biosciences, University of Milano-Bicocca, 20126 Milan, Italy

**Keywords:** psoriasis initiation, environmental trigger, inflammasome activation, purinergic signaling, P1 receptors, P2 receptors, psoriasis pathogenesis

## Abstract

Psoriasis is a chronic inflammatory disease of the skin associated with systemic and joint manifestations and accompanied by comorbidities, such as metabolic syndrome and increased risk of cardiovascular disease. Psoriasis has a strong genetic basis, but exacerbation requires additional signals that are still largely unknown. The clinical manifestations involve the interplay between dendritic and T cells in the dermis to generate a self-sustaining inflammatory loop around the TNFα/IL-23/IL-17 axis that forms the psoriatic plaque. In addition, in recent years, a critical role of keratinocytes in establishing the interplay that leads to psoriatic plaques’ formation has re-emerged. In this review, we analyze the most recent evidence of the role of keratinocytes and danger associates molecular patterns, such as extracellular ATP in the generation of psoriatic skin lesions. Particular attention will be given to purinergic signaling in inflammasome activation and in the initiation of psoriasis. In this phase, keratinocytes’ inflammasome may trigger early inflammatory pathways involving IL-1β production, to elicit the subsequent cascade of events that leads to dendritic and T cell activation. Since psoriasis is likely triggered by skin-damaging events and trauma, we can envisage that intracellular ATP, released by damaged cells, may play a role in triggering the inflammatory response underlying the pathogenesis of the disease by activating the inflammasome. Therefore, purinergic signaling in the skin could represent a new and early step of psoriasis; thus, opening the possibility to target single molecular actors of the purinome to develop new psoriasis treatments.

## 1. Introduction

Psoriasis is a chronic inflammatory disease of the skin associated with systemic and joint manifestations and accompanied by comorbidities, such as metabolic syndrome and increased risk of cardiovascular disease [1,2]. The disease has a strong genetic basis, but its exacerbation requires additional signals that are still under investigation. The immune mechanisms leading to the amplification of skin inflammation, and possibly to systemic manifestations, have been characterized in detail in the last two decades and involve the interplay between innate and adaptive immunity to generate a self-sustaining inflammatory loop. This is sustained by TNFα, IL-23, and IL-17, which are pivotal cytokines in generating and maintaining the psoriatic plaque [3]. Adaptive immune responses in psoriasis patients include an autoimmune component made by T cells recognizing or cross-recognizing self-antigens [4,5,6]. Despite this growing knowledge, the early mechanisms paving the way to psoriasis manifestations still remain largely unknown. In particular, the sequence of events preceding the appearance of symptoms needs to be further elucidated.

In this perspective, the role of keratinocytes as key upstream elements in the psoriatic inflammatory cascade has recently been reassessed [7,8].

Indeed, psoriasis was originally considered a pathology of keratinocytes and these cells were seen as the main drivers of the disease. However, in 1979, cyclosporine was found to effectively treat psoriasis by inhibiting the cytokines produced by T cells [9]. In addition, a human skin transplantation mouse model showed that psoriasis manifestations could be transferred with T cells, showing T-lymphocyte dependence of the pathology and leading to the current classification of psoriasis as an immune-mediated disease [10].

More recently, the role of skin epidermis in the early pathogenic steps of psoriasis has re-emerged and its contribution to the activation of the immune response, as well as to the recruitment of inflammatory and endothelial cells, has been clearly demonstrated [7,11,12,13].

In addition, the extended characterization of the psoriasis susceptibility genes, obtained by genome-wide association studies (GWAS), has allowed a broader, detailed understanding of the main mechanisms of the pathogenesis, underlining the importance of epidermis and keratinocytes-mediated inflammatory pathways in triggering psoriasis [14].

PSORS1 is known as the main psoriasis susceptibility locus. It is located on chromosome 6, and within this locus, the HLA-C∗06:02 allele is the major genetic determinant, accounting for 50% of psoriasis heritability. Other gene variants associated with psoriasis include genes belonging to the IL-23/IL-17 axis, as well as genes involved in CD8^+^ T differentiation, antigen processing [15], NF-kB/IL-1/TNFα production, and in type-I interferon-mediated responses [16]. Single-nucleotide polymorphisms (SNPs) associated with psoriasis were found in genes encoding transcription factors, such as REL, TYK2, STAT3, or RUNX3 [17,18].

*PSORS4* is another psoriasis susceptibility locus, located in chromosome 1q21. This region contains genes of the epidermal differentiation complex (EDC). EDC genes are divided into 3 main families encoding cornified envelope precursor proteins, keratin filament-associated proteins, and the S100 calcium-binding proteins [19,20].

It is interesting to point out that in other psoriasis variants, such as generalized pustular psoriasis, an association with mutations in *CARD14* and *IL36RN* genes, which are involved in the NF-κB pathway and in IL-36 cytokine signaling, has been shown [21].

IL-36RN is a protein that acts as an antagonist of the activity of the IL-1 family cytokine IL-36 by binding to its receptor and preventing signaling. The consequence of mutations within the *IL36RN* gene is an increased transcription of NF-κB-regulated genes. *CARD14* is expressed by keratinocytes and endothelial cells; gain-of-function mutations in this gene lead to increased activation of NF-κB [22].

In this updated view, intrinsic alterations in epidermal keratinocytes could represent a relevant link to the dysregulation of the major inflammatory pathways, such as the NF-κB signal transduction pathway, promoting the production of TNF-α, IL-1β, IL-6, and IL-12p40 key inflammatory cytokines and CCL2, CCL5, CXCL2, and CXCL10 chemokines [23,24,25].

In this review, we would like to analyze the most advanced evidence of the role of keratinocytes and danger-associated molecular patterns, such as extracellular ATP and purinergic signaling, in inflammasome activation and psoriasis initiation. In particular, we will consider the possibility that an insult damaging keratinocytes may trigger the inflammatory pathways involving inflammasome activation and IL-1β production to mediate the subsequent cascade of events that lead to T cell activation by dendritic cells (DC), underlying the development of psoriatic plaques.

## 2. Immunopathogenesis of Psoriasis and the Regained Role of Keratinocytes

T cell-mediated psoriasis is likely to occur down-stream of early triggering events, which take place in a subclinical and asymptomatic phase of the disease [6].

According to the current view, immuno-mediated pathogenesis of psoriasis starts with an external trigger that leads to LL-37 production and self-DNA release by injured keratinocytes. LL-37, conjugated with self-DNA, is taken-up by myeloid DC (mDC) and plasmacytoid DC (pDC), in which DNA leads to the stimulation of TLR9, while RNA motifs stimulate the toll-like receptors (TLR) 7 and 8. TLR7/8-stimulates mDC to secrete TNFα, IL-23, and IL-12, while pDCs produce large amounts of IFNα [4].

Skin injury also activates TLR3 in keratinocytes, leading to the production of proinflammatory cytokines, such as TNFα, IL-6, and IL-36 [26,27]. In addition to injury, mechanical stretch is also capable of inducing the production of inflammatory cytokines [28].

This emerging evidence points towards a prominent role for keratinocytes in the pathogenic mechanisms responsible for the onset of psoriasis [29]. In the early phase, it is likely that keratinocytes may react to damage or bacterial triggers, with an initial release of inflammatory cytokines and chemokines that finally leads to the activation of the self-sustaining inflammatory loop.

In psoriatic skin lesions, the CD11c^+^ inflammatory myeloid DC expressing TNFα, IL-23, and iNOS (TNF and iNOS-producing TIP-DC) are present at a higher frequency and can be activated by alarmins produced by keratinocytes and neutrophils, to finally stimulate pathogenic T cell responses [30]. The primary activation of pathogenic T cells occurs in the lymph nodes, where DC prime differentiation of naive T cells through IL-1β, IL-6, and IL-23 into Th17/Tc17 cells, whereas IL-12 polarizes T cells towards a Th1/Tc1 phenotype [1,31,32].

IL-17 can act on keratinocytes to induce the production of chemokines, cytokines, and antimicrobial peptides that recruit neutrophils, macrophages, and more T cells to the site of inflammation. Moreover, cytokines also directly act on keratinocytes to regulate their proliferation and differentiation, culminating in a positive feedback loop [33].

Keratinocytes participate with psoriasis pathogenesis through the production of IL-1 family cytokines and the constitutively active proinflammatory NF-κB pathway.

In addition, IL-36γ, which is strongly upregulated in psoriatic plaques, likely plays a pivotal role in the disease [34,35,36].

Importantly, a recently published review introduced the “epithelial barrier hypothesis”. This hypothesis underlines the crucial role of skin and mucosal barriers in the protection from environmental stress and in the maintenance of tissue homeostasis, addressing the possibility that an altered epithelial barrier function may predispose to allergic and autoimmune conditions [37]. Although this concept mainly refers to the role of microbial dysbiosis, it can be postulated that environmental stressors could also affect epithelial barrier function through other mechanisms (that may include epigenetic modifications) leading to the expression of inflammatory genes.

The role of epidermal barrier permeability in psoriasis is supported by the association with *PSORS4* genes encoding proteins involved in epidermal differentiation and function. In addition, it has been shown that the deletion of the proteins, associated with differentiation, also leads to an upregulation of cytokines and inflammatory genes [38].

Healthy microbiota on the epithelial barrier can regulate the barrier homeostasis at the levels of permeability, local microinflammation, and immune-regulation. As a consequence, the reduced biodiversity of skin microbiota may promote different inflammatory conditions [39,40,41,42,43].

## 3. Alarmins/DAMPs in Psoriasis Pathology

Initial triggering events of psoriasis include streptococcal infections, traumas, and medications [44]. Dysbiosis of the skin microbiome in individuals with psoriasis has been reported. Changes were mainly observed in the relative abundance of Firmicutes, Actinobacteria, and Proteobacteria; however, *Staphylococcus* and *Streptococcus* spp. have also been reported to be more abundant in psoriatic skin [39]. The association between streptococcal infections is well established for guttate psoriasis (56–97% association) and has recently been extended to the exacerbation of plaque psoriasis [40,45,46]. As a possible mechanism to explain this association, is the evidence that streptococcal superantigens may favour the activation of T cells but also of keratinocytes expressing HLA-DR molecules in pathological conditions [47,48].

On the other hand, commensal bacteria present in healthy skin may exert immune regulatory functions. Therefore, the reduction of commensal species and colonization by non-commensal bacteria may favour inflammatory reactions and impaired barrier functions.

As seen in a mouse model of atopic dermatitis, the-enhanced exposure to components of the skin microflora that trigger keratinocytes TLRs may induce the skin inflammatory process [41]. TLR signaling could also be also relevant for the priming phase of inflammasome assembly that includes the transcription of inflammasome genes [42]. Of note, TLR triggering is favoured by alarmins, such as antimicrobial peptides, cathelicidins, β-defensins, and S100 proteins, which are increased in psoriatic skin [43].

Alarmins are endogenous molecules produced by keratinocytes that, once released in the extracellular environment, can inactivate bacteria. They can, therefore, play a role in linking an innate mechanism of protection against bacterial infections and a stimulation of the inflammatory response that could contribute to the initial phase of psoriasis.

S100 family proteins are important in the pathogenesis of psoriasis. IL-17A has been shown to stimulate the production of S100A7 (psoriasin) and S100A15 (koebnerisin) by keratinocytes [49]. The calgranulins S100A8 and S100A9 are produced by myeloid cells and keratinocytes and are released during cellular stress, forming heterodimeric S100A8/S100A9 complexes markedly overexpressed in psoriatic skin [49]. Among alarmins, human β-defensins 2 and 4 are known to bind DNA and stimulate pDC in a TLR9-dependent manner [43]. Therefore, alarmins can favor the binding of self-DNA and self-RNA (danger-associated molecular patterns) to their receptors and favor the secretion of interferon-α from keratinocytes and pDC, thus, inducing inflammation responses.

Cutaneous manifestations of psoriasis actually appear in areas of injury (Koebner phenomenon) and plaques usually initiate on elbows and knees (areas commonly exposed to trauma).

**Figure 1:**
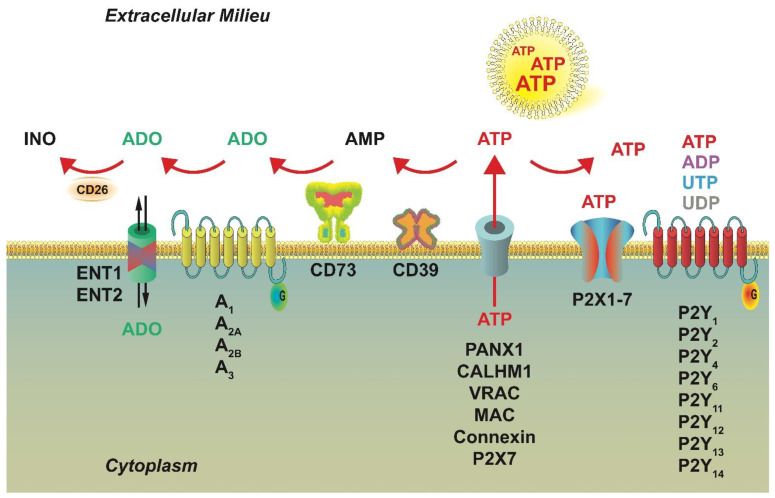
Purinergic signaling molecules. Intracellular adenine (ATP and ADP), uridine nucleotides (UTP and UDP), and nucleosides (ADO) play the role of cell-to-cell mediators once released, as a consequence of shear stress membrane damage, hypoxia, apoptosis, necrosis, and infections. They can be transported by specialized molecules (PANX1, CALHM1, VRAC, MAC, Connexin, and P2X7) and act as agonists at purinergic P2 (P2X and P2Y) or P1 (A_1_, A_2A_, A_2B_, and A_3_) receptors. ADO, which activates P1 receptors, can also be transported extracellularly or generated on the outer surface of the cell by the enzymatic conversion of ATP/ADP to AMP by the ectonucleoside triphosphate diphosphohydrolase CD39 and with the hydrolysis of AMP to ADO by the ecto-5′-nucleotidase CD73.

Traumatic tissue damage and cellular shear stress can induce the release of “danger-associated molecular patterns” (DAMPs) or danger signals among them extracellular ATP, whose cytoplasmic concentration is around 5–10 mM (close to 0 outside the cell). An increase in the extracellular ATP concentration is perceived by the surrounding cells as a DAMP, inducing activatory and proinflammatory responses [50,51]. Besides plasma membrane damage, ATP can be transferred outside the cell by different means, among which membrane molecules, such as ATP-binding cassette transporters, connexins, pannexin 1, P2X7, and vesicular exocytosis (Figure 1). Extracellular synthesis of ATP via plasma membrane F(1)/F(0)-ATP synthase is also possible.

Hence, uncontrolled or prolonged ATP release causes excessive immune cell activation, leading to increased secretion of proinflammatory cytokines, prostaglandins, leukotrienes, reactive oxygen intermediates (ROIs) and granular enzymes, causing tissue damage and attracting immune cells with the establishment of chronic inflammation. Moreover, the nucleoside adenosine (ADO) derived from extracellular ATP hydrolysis, or by cytoplasm extrusion, favours the keratinocyte proliferation and turn-over that are found in psoriatic skin [52].

Since psoriasis often starts at the site of physical trauma [53], ATP has to be necessarily released extracellularly by keratinocytes, melanocytes, and/or other damaged cells. Once in the extracellular milieu, the nucleotide may play different roles both by inducing chemotaxis of immune cells and by amplifying the inflammatory response [54].

## 4. The Purinergic Signaling

As extracellular mediators of cell-to-cell communication, ATP, ADP, UTP, UDP, and the nucleoside adenosine (ADO) activate specific cell-surface molecules, i.e., purinergic P2 and P1 receptors, respectively (Figure 1), modulating tissue responses, such as cell metabolism, secretion of cytokines and chemokines, production of inflammatory mediators, matrix deposition, cell proliferation, and even cell death. Availability of extracellular nucleotides and ADO in the tissue *milieu* is dependent on their release from the cytoplasm, as well as by the activity of membrane kinases, nucleoside and nucleotide transporters, ectonucleotidases ENTPD1 (CD39), and 5′-NT (CD73).

P1 or ADORA receptors comprise of A_1_ (ADORA1), A_2A_ (ADORA2A), A_2B_ (ADORA2B), and A_3_ (ADORA3) subtypes. They are G-protein-coupled receptors with seven-transmembrane domains and are able to modulate adenylate cyclase activity (AC). A_2A_ and A_2B_ activate the enzyme, while A_1_ and A_3_ inhibit it [55]. They also show a difference in ADO affinity as A_1_, A_2A_, and A_3_ receptors are activated by lower ADO concentrations than A_2B_ (10–50 nM and 1 mM ADO, respectively) [56]. ADO is transported through the cell membrane by specialized transporters (Figure 1). Nucleoside concentration increases extracellularly during hypoxia or inflammation [57]. Degradation of extracellular ATP by ectonucleotidases increases ADO concentration in tissue *milieu*.

Ectonucleotidases transforming ATP to ADP, and then to ADO, produce the agonist of P1 receptors, via a sequence of degradative reactions [58,59,60]. These enzymes are central players in avoiding accumulation of extracellular signaling for P2 receptors, thus, preventing the development of conditions that favour immuno-mediated tissue damage and autoimmune diseases [57].

P2 receptors are activated by extracellular nucleotides and subdivided into two subgroups, P2X and P2Y [61] (Figure 1).

P2Y receptors are seven-transmembrane domains and couple intracellularly to Gq/G11 or Gi/0 proteins; they include eight subtypes: P2Y_1_, P2Y_2_, P2Y_4_, P2Y_6_, P2Y_11_, P2Y_12_, P2Y_13_, and P2Y_14_. P2Y receptors have multiple agonists (Figure 1) being activated by different adenine and uridine nucleotides. P2Y_1_, P2Y_12_, and P2Y_13_ subtypes are mainly activated by ADP, P2Y_2_ is activated by UTP or ATP, and finally, P2Y_4_ and P2Y_11_ by UTP and ATP, respectively; P2Y_6_ by UDP and P2Y_14_ by UDP-glucose. P2Y receptors modulate several physiological responses [62,63].

The ATP-gated, trimeric P2X receptors comprise of seven subtypes (P2X1−P2X7) that can assemble giving homo- or hetero-trimers. They are ion channels, selective for monovalent and divalent cations, such as Na^+^, K^+^, Ca^2+^, and Mg^2+^; their activation evokes transmembrane ion fluxes, modulating several signaling cascades [64]. A particular interest was devoted to P2X7 for its capacity of modulating the production and secretion of inflammatory cytokines (IL-1β, IL-18, IL-6) [65,66]. The importance of this receptor in building the inflammatory response is confirmed by knocking-down its expression. The lack of P2X7 functionality greatly attenuated, or even abolished, the production of pivotal cytokines, fuelling the inflammatory background in animal models of atherosclerosis, Alzheimer’s disease, and allergy [67]. P2X7 signaling in antigen-presenting DC has been shown to increase the expression of CD80 and CD86 and activate a cascade of proinflammatory events, including STAT1 phosphorylation and IFNγ production. Other reports also indicated that P2X7-mediated signaling could contribute to the differentiation of Th17 responses [66].

## 5. The Inflammasome

Increasing evidence enlightens the importance of keratinocytes as sensors of danger, exerting this role through alert systems, such as the inflammasome. Inflammasome is a large multiprotein complex formed by a sensor, an adaptor protein present in the majority of the structures (ASC also known as PYCARD), and an effector (caspase 1). There are different types of sensors that characterize different inflammasomes. These include a family of NOD-like, receptor-pyrin-containing proteins (NLRP) and the interferon-inducible AIM2 and IFI16 sensors that recognize cytosolic DNA through their hematopoietic, interferon-inducible nuclear (HIN) domain.

NLRP3 is an intracellular sensor that detects a broad range of microbial motifs, endogenous danger signals, and environmental irritants, finally leading to caspase 1-dependent release of the pro-inflammatory cytokines IL-1β and IL-18, as well as to gasdermin D (GSDMD)-mediated pyroptotic cell death [42].

NLRP3 inflammasome requires priming and activation. The priming signal, provided by cytokines or pathogen-associated molecular patterns (PAMPs), leads to the transcriptional upregulation of canonical and non-canonical NLRP3 inflammasome components.

The second signal is provided by numerous PAMPs, or DAMPs, such as particulates, crystals, and extracellular ATP, through P2 receptor-activation evoking K^+^ efflux, Ca^2+^ influx, lysosomal disruption, mitochondrial reactive oxygen species (mtROS) production, and the relocalization of cardiolipin to the outer mitochondrial membrane; the release of oxidized mitochondrial DNA (Ox-mtDNA) NLRP3 is activated by infective agents, as well as, in sterile inflammation, by endogenous DAMPs, such as ATP, cholesterol and monosodium urate crystals, calcium pyrophosphate, neutrophil extracellular traps, cathelicidin, and oxidized mitochondrial DNA. Xenobiotics such as alum, silica, and imiquimod, which is used for the induction of psoriasis-like-inflammation in the mouse modelcan also activate NLRP3 [42].

It has recently been established that ultraviolet irradiation activates NLRP3 in human keratinocytes [68,69]. In addition, contact sensitizers, such as haptens, applied to the skin, induce inflammasome-dependent IL-1β and IL-18 processing and secretion [23].

## 6. Inflammasome in Psoriasis

Inflammasome activity has been implicated in several chronic inflammatory disorders [25,70,71,72] and new evidence in the field of barrier tissues also points towards a possible role in the pathogenesis of psoriasis [73,74]. An early study in this field has shown a 20-fold increase of caspase-5 mRNA expression and a moderate, but significant, increase of caspase 1 and other inflammasome-related transcripts in psoriatic lesions [75]. Increases of NLRP3, NLRP1, and AIM2 in psoriatic skin samples have been reported by different studies that have been exhaustively summarized by Ciążyńska et al. in a review article in this Special Issue [74,76,77,78,79]. Along this line, genetic data have also indicated an association with polymorphism in *NLRP1*, *NLRP3*, and *AIM2* in psoriasis [76,77,78,80,81,82], and more recently, the association with a genetic polymorphism in inflammasome-related genes has been shown in patients with psoriatic arthritis [82]. In addition, increased expression of NLR-signature genes has been reported, both in full thickness skin samples and in psoriatic epidermis samples [83,84,85,86,87].

A very recent study reported the enhanced activity of inflammasome in the peripheral blood of patients with psoriasis and proposed this mechanism as a link between psoriasis and systemic inflammation. The study shows that patients with psoriasis have increased plasma levels of IL-1β and IL-18 and increased constitutive expression of the inflammasome sensors *NLRP3*, *NLRP1*, and *AIM2* in peripheral blood cells, together with increased caspase 1 reactivity [88]. Importantly, the activation of IL-1β was linked to the increased production of IL-17 by T cells in both humans and models [89,90].

In mouse models, TNF-α has been shown to regulate the transcription of NLRP3 inflammasome components [91] and exposure to TNF-α results in the significantly increased expression of *NLRP3* and *pro-IL-1β*. The study showed that TNF-α can directly activate NLRP3 inflammasome, without the requirement for a priming signal, and that patients undergoing treatment with TNF-α blocking agents had normalized plasma IL-1β and IL-18 levels, as well as normalized caspase 1 reactivity [92].

A study by Tervaniemi and colleagues specifically focused on the differential gene expression in the epidermal samples. This analysis evidenced that NOD-like receptors signaling and inflammasome-related pathways were increased, as compared to control skin or to non-lesional skin [83]. In particular, the expression of genes encoding components of NOD-like receptor (NLR) signaling, RIG-like receptor (RLR) signaling, and cytosolic DNA sensing pathways were markedly increased in psoriatic epidermis. The differentially expressed genes of NLR signaling included highly upregulated transcripts: *NOD2*, *CARD6*, *CARD18*, *CASP5*, *IL1B*, *IL8*, and *CXCL1*, as well as other components, such as *NLRP10*, *NLRX1*, *CASP1*, *CASP8*, and the adaptor protein PYCARD. Transcripts for the receptors of the cytosolic DNA sensing pathway and RLR pathways included DNA-binding receptors *AIM2* and *IFI16* and RNA helicase proteins *IFIH1* and *DDX58* (*RIG-I*), as well as other RLR-related transcripts [83]. This evidence strongly reinforces the possibility that inflammasome activation in keratinocytes is involved in psoriasis pathogenesis.

Cytosolic DNA has been identified as a danger signal that activates AIM2 inflammasomes. In cultured keratinocytes, AIM2 and cytosolic DNA triggered the release of IL-1β, therefore indicating that cytosolic DNA can trigger AIM2 inflammasome and IL-1β activation in psoriasis [93]. Strikingly, a recent work by Naik et al. in a mouse model demonstrated, that upon tissue damage or imiquimod treatment, the epithelial stem cells of the skin epithelium basal layer acquired long-term memory of an inflammation by increased open chromatin domains. These open-chromatin domains persisted to inflammation and contained the information to sense tissue damage, such as the gene encoding AIM2. Upon a secondary challenge, genes of these domains were rapidly transcribed. The authors showed that the lack of *Aim2*, or the inhibition of its effectors (caspase 1 and IL-1β), abolished the capability to recall inflammation. In this pathological context, the enhanced sensitivity to develop inflammation upon a primary exposure could possibly increase susceptibility to autoimmune and hyper-proliferative disorders. Moreover, memory acquisition, through increased chromatin accessibility of inflammasome genes, could contribute to the recurrence of psoriasis [94,95].

These concepts together may open to the possibility that constitutive increase of inflammasome activity can favor the establishment of the self-sustaining, proinflammatory loop in the skin of a genetically predisposed individual upon external triggering with environmental stressors, through a mechanism that may involve epigenetic modifications.

## 7. Purinergic Signaling in Inflammasome Activation and in Disease Pathogenesis

Modulation of purinergic signaling regulates many skin functions, including keratinocyte proliferation, skin repair, immune response, matrix deposition, and fibrosis. The release of ATP by damaged cells has been widely documented; however, in physiological conditions, the nucleotide can be transferred extracellularly by means of specialized membrane molecules (Figure 1). These include the connexin hemichannels (CX), pannexin 1 channel (PANX1), calcium homeostasis modulator 1 (CALHM1), volume-regulated anion channel (VRAC), and maxi-anion channel (MAC) [96]. These molecules participate with the onset of inflammation by modulating the interaction between the immune and endothelial cells, favouring extravasation; furthermore, they stimulate the release of signaling molecules, creating proinflammatory conditions by activating leukocytes in an autocrine manner [97]. ATP can induce the long-term activation of immune cells, leading to chronic inflammatory conditions often accompanied by tissue damage and fibrosis. CX are widely distributed throughout the epidermis, where they play different roles [98]. The Reciprocal activation of CX and purinergic receptors has been shown [96]. Extracellular ATP stimulates P2Y receptors that, through intracellular mediators, in turn, activate the release of the nucleotide through the CX molecule.

**Figure 2:**
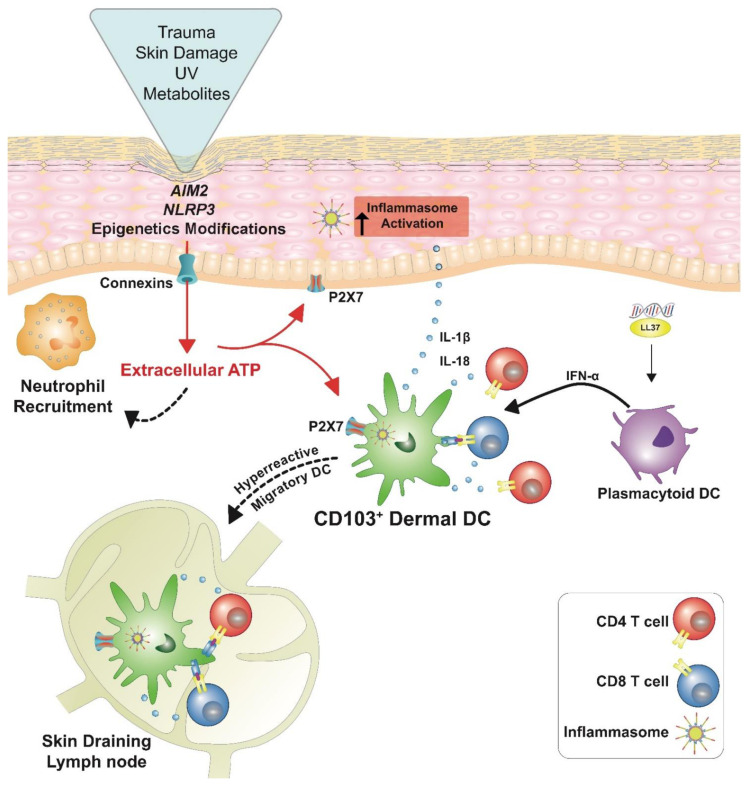
Hypothetical model for the early phase of psoriasis. An initial skin damaging event, caused by mechanical trauma, UV irradiation, or the accumulation of cell metabolites in the presence of susceptibility gene variants or epigenetic modifications, induce ATP release, both by cell membrane damage and connexin activation. Extracellular ATP may act as a chemotactic stimulus for immune cells and activate the inflammasome by binding the P2X7 receptor in keratinocytes and in CD103^+^ dermal DCs. This stimulates the production of IL-1β and IL-18 and hyper migration of CD103^+^ dermal DCs to the skin draining lymph-node, where they present antigens to CD4^+^ and CD8^+^ T lymphocytes.

This evidence has prompted the idea that an excessive release of ATP, as a consequence of skin traumatic events, radiations, infections, immunologic attack, or the accumulation of metabolites (Figure 2) may evoke an auto-amplifying, CX-mediated ATP release, leading to skin inflammation and the attraction of immune cells into the epidermis [99,100].

It was nicely shown that ATP and the pro-inflammatory cytokine IL-6 were released by human keratinocyte cells (HaCaT) challenged with staphylococcus peptidoglycan. Connexin involvement was confirmed by CX-channel blockers, which inhibited both ATP and IL-6 secretion, while IL-6 was decreased by purinergic blockers [100].

CX26 modulates the epidermal barrier and remodelling of the skin during wound healing [101]. In mice, the sustained expression of CX26-induced epidermal hyperproliferation blocked the transition to tissue remodelling and caused the infiltration of the inflammatory cells, finally leading to the development of psoriasis-like inflammation [101]. Mechanistically, the ectopic expression of CX26 in keratinocytes resulted in increased ATP [102]. The expression of this connexin undergoes a huge increase in psoriatic patients. Strikingly, CX26 transcription and translation were potently up-regulated (>100×) in the psoriatic and non-psoriatic tissue of psoriasis patients, but not in normal controls. Another relevant observation was that CX43 expression was unchanged, although CX43 protein was found post-translationally modified and accumulated in psoriatic tissue [103]. Although it is not possible to directly link excessive CX-induced ATP released to the proinflammatory background found in psoriasis, it is peculiar that fibroblasts from psoriatic subjects showed a higher inflammatory index, as compared to normal ones, and they drove normal keratinocytes towards a “psoriatic phenotype” [3,103,104]. Experimental evidence in mice shows that purinergic signaling has the ability to modulate IL-17 and IL-23 release [105,106]. In particular, DCs obtained from bone marrow of P2Y_12_^−/−^ mice undergoing experimental autoimmune encephalomyelitis secreted higher amounts of IL-23, which is fundamental to induce differentiation of CD4^+^ T lymphocytes towards the pathogenic Th17 IL-17A [106]. In these mice, pathological changes were also accompanied by an increased number of Th17 lymphocytes and augmented sera concentration of IL-17A [106]. However, in another report on P2Y_12_^−/−^ mice, authors found less brain infiltration by leukocytes and decreased IL-17 expression [105]. In C57BL/6 mice, the P2Y_12_ antagonist Clopidogrel inhibited Th17 differentiation [105].

ADO has also been involved in changes present in psoriatic skin [107,108]. The nucleoside, which is an agonist at P1 receptors, although with a different concentration requirement, has been indicated as an important player in tissue changes, such as collagen production and the stimulation of proliferation in epidermis. Moreover, ADO plays an anti-inflammatory role in skin and other tissue, through the activation of the A_2A_ receptor subtype. The topical application of the A_2A_ agonist CGS-21680 was shown to prevent the epidermal hyperplasia and inflammation in Swiss CD-1 mice challenged with phorbol [109]. In C57BL/6 mice that underwent experimental autoimmune encephalomyelitis, the same A_2A_ agonist caused a relevant proliferation to decrease in Th1, Th2, and Th17 lymphocytes, while it increased Treg numbers [110], suggesting that A_2A_ receptor subtype mediates immunosuppression in different tissues and experimental models. The A_2B_ subtype seems to have a pro-fibrotic effect as dermal fibrosis, induced by bleomycin, in mice was reduced by the A_2B_ selective blocker GS-6201 [111]. Finally, A_3_ receptors were found to be highly expressed in inflamed tissues from animal models of adjuvant-induced arthritis, as well as in peripheral blood mononuclear cells of patients with rheumatoid arthritis [112]. Additionally, A_3_ receptors were found to be overexpressed in peripheral blood mononuclear cells from patients with psoriasis [113]. A randomized clinical trial showed that A_3_ receptor stimulation with the CF101 agonist improved the clinical symptoms of psoriasis [114]. Moreover, a synergistic effect between methotrexate and CF101 has been reported. As a possible mechanism explaining this phenomenon, it has been proposed that methotrexate can induce an increase in A_3_ receptor expression in inflamed tissues, which may increase the tissue responsiveness to the treatment with the A_3_ agonist CF101 [115].

In psoriasis patients, ATP release has been reported in association with Koebner phenomenon and it is released in vitro by stretched keratinocytes. It is, therefore, possible that ATP release and purinergic signaling can represent one of the mechanisms involved in psoriasis exacerbation and/or reactivation [116]. In support of this view, it has been reported that P2X7 and P2Y_1_ receptors are upregulated in lesional skin of patients with psoriasis. An increase in P2X7 receptor expression was also found in nonlesional skin of psoriatic patients, in comparison with healthy skin tissue, leading the authors to hypothesize that P2X7 receptor dysregulation in psoriasis precedes the onset of inflammatory lesions. In human skin explants, the pharmacological stimulation of P2X7 with the potent pharmacological agonist BzATP, induced a significant increase in vascular endothelial growth factor (VEGF), IL-23 and IL-6 expression, suggesting that P2X7 activation may be an initiating factor in psoriasis development [117].

The cutaneous immune responses, induced upon P2X7 stimulation, have been demostrated to be, at least in part, dependent on inflammasome/IL-1β pathways. In particular, VEGF, iNOS and S100A7 appear to be downstream of the inflammasome pathway, whereas PGE_2_ would be produced by an inflammasome-independent mechanism, mediated through intracellular calcium influx and MAPK signaling. Similarly, CXCL10 and TNFR1 increased early in the skin upon P2X7 activation, possibly through MAPK signaling. Inflammasomes are now recognized as critical innate immune components that orchestrate host immune responses, including antigen presentation and T cell polarization [54]. P2X7 signaling is indeed capable of activating the NOD-like-receptor-mediated inflammasome assembly, pro-caspase 1 proteolytic activation and pro-IL-1 and pro-IL-18 cleavage and release of biologically active IL-1β and IL-18 [118]. Upon tissue damage, dying cells release DAMPs, including ATP that binds P2X7 receptors on DC. This could lead to activation of the NLRP3 inflammasome by the ATP/P2X7axis and to production of IL-1β that can induce CD8 T responses via IL-1 receptor (Figure 2). The role of NLRP3 inflammasome activation on has been reported for the induction of primary response in antitumor immunity through the induction of a hyperreactive state in dendritic cells that favors migration to the lymph node and cross-presentation of antigens from damaged cells [54,119]. This mechanism could be extended to any CD8 T cell-mediated response, including that directed towards self-antigens developing in psoriasis patients.

## 8. Conclusions

Psoriasis is a diffuse skin pathology with heavy psychological and economic consequences. The initial steps of psoriasis, as well as their link with the environmental triggers and with the downstream cascade of immune events leading to psoriasis manifestations are still unknown. Moreover, no treatment is available to cure the disease at its onset or to prevent the development of systemic and extra cutaneous manifestations, such as psoriatic arthritis. Inflammasome genes are highly upregulated in psoriatic epidermis and the evidence collected here points out the roles of AIM2 an NLRP3 in the pathogenesis of psoriasis and its systemic manifestations. Most importantly, the evidence of chromatin modifications that occur upon tissue damage and inflammation increase *Aim2* gene accessibility and transcription, conferring to the epidermal keratinocytes with sternness, feature the long-term memory of inflammation may pave the way to a new interpretation of the initiation phase of psoriasis and of its recurrence. In this context purinergic signaling can play a relevant role at different levels in the skin. In particular, P2X7, through its binding to extracellular ATP, may represent an effective stimulus for inflammasome activation in both keratinocytes, to start the inflammatory process, and dermal DC to increase migration to lymph nodes for antigen presentation. Therefore, inflammasome activation by P2X7 signaling may represent not only a potential step in the initiation phase of psoriasis, but it could also provide a link with downstream inflammation and generation of the autoimmune component of the disease.

Recent investigations have shown that inhibitors of the ADO degrading enzyme, adenosine deaminase (ADA) were able both in IL-10^−/−^ where they caused a decrease in Th1 cytokines (IL-1β, IFN-γ, TNF, IL-6 and CXCL10) and ameliorated the clinical and histologic score in severe colitis [120], but also in cancer patients with hairy cell leukaemia. In this latter case, they induced a stable resolution of severe psoriasis, opening the concrete possibility of targeting extracellular ADO degradative pathways, as well as P2X7 receptor as new therapeutic options to treat psoriasis at its onset and possibly recurrence [121,122].

## Data Availability

Not applicable.
